# Retrospective Review on Reticular Materials: Facts and Figures Over the Last 30 Years

**DOI:** 10.1002/adma.202414736

**Published:** 2025-05-15

**Authors:** Aamod V. Desai, Stefano Canossa, Ekaterina A. Chernova, Simon M. Vornholt, Konstantin Stracke, Jack D. Evans, E. Eja Petersen, Stefan Wuttke, Romy Ettlinger

**Affiliations:** ^1^ EaStCHEM School of Chemistry University of St Andrews North Haugh St Andrews KY16 9ST UK; ^2^ Department of Chemistry and Applied Biosciences ETH Zürich HCI H 103, Vladimir‐Prelog‐Weg 1–5/10 Zürich 8093 Switzerland; ^3^ Basque Center for Materials Applications and Nanostructures (BCMaterials) Bld. Martina Casiano, 3rd Floor UPV/EHU Science Park Barrio Sarriena s/n Leioa 48940 Spain; ^4^ Department of Chemistry Stony Brook University Stony Brook New York 11794 USA; ^5^ School of Physics Chemistry and Earth Sciences The University of Adelaide North Terrace Adelaide SA 5005 Australia; ^6^ Academic Centre for Materials and Nanotechnology AGH University of Krakow Krakow 30‐059 Poland; ^7^ TUM School of Natural Sciences Department of Chemistry Technical University of Munich Lichtenbergstrasse 4 85748 Garching Germany

**Keywords:** covalent organic frameworks, databases, facts, metal–organic frameworks, milestones, reticular materials, survey

## Abstract

The field of reticular materials, such as metal–organic frameworks (MOFs) and covalent organic frameworks (COFs), is expanding continuously – be it in terms of novel structures, advanced characterization techniques, or record‐breaking physical properties for applications. This timeline review reflects on the progress over the past 30 years, complemented by input from the community of active researchers. Owing to a global, crowdsourced survey of 228 researchers that is conducted through an online questionnaire, recent insights into the demographics of the field are given. Besides revealing how it works, publish, and interact, the review highlights both academic and industrial milestones. The contemporary trends are described, both at the level of material development and their suitability for a range of applications. To pave the way for newcomers to the field, some remaining challenges and steps to overcome them are discussed. The findings from this contemplative review aim to shape the future course of research in this domain.

## Graphical abstract

To shape the future course of research in reticular materials, this work reflects on the progress over the past 30 years, complemented by input from the community of 228 active researchers through a global, crowdsourced survey: ranging from demographics, how it works, publish and interact, to highlights on both academic and industrial milestones, as well as remaining challenges.

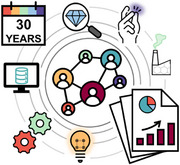

## 1 Introduction

**Figure 1:**
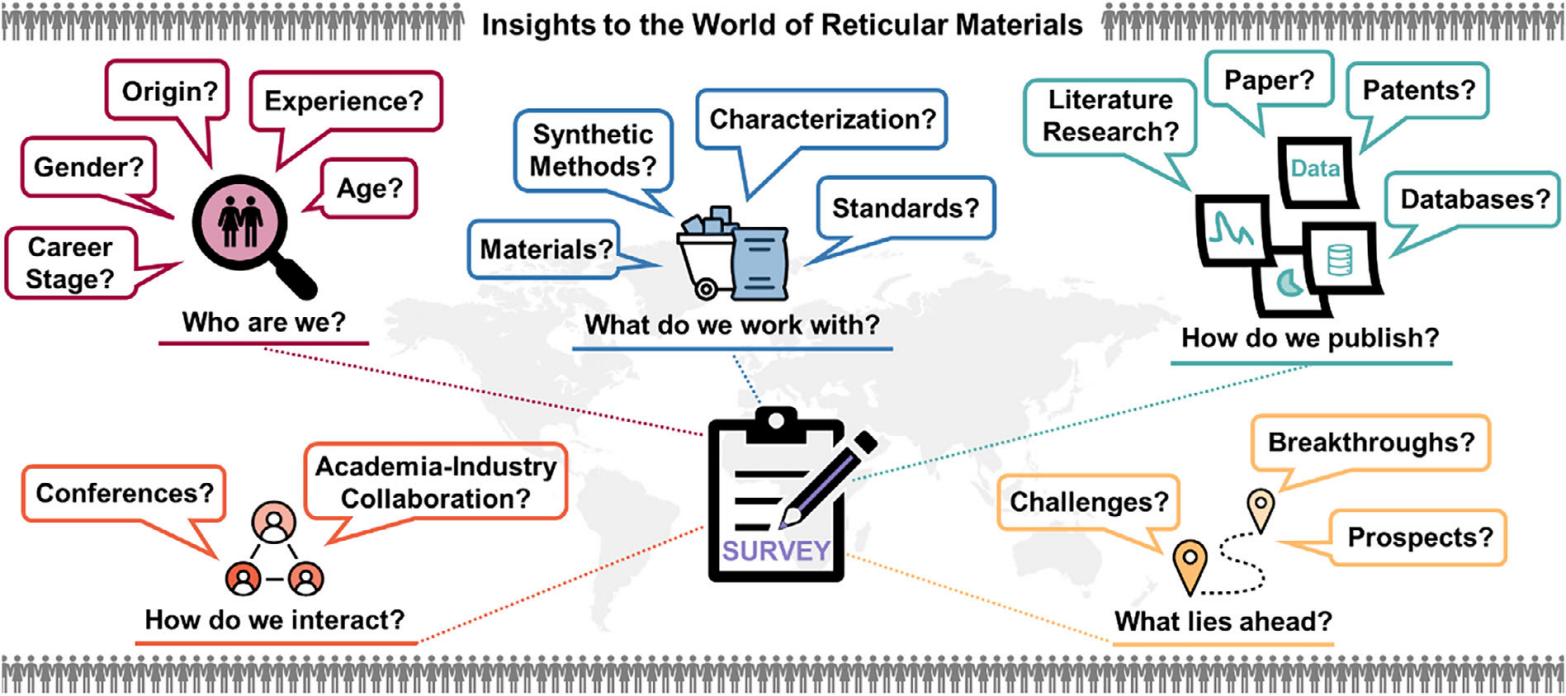
Overview of the relevant survey topics of this review to gain insight into the world of reticular material.

Reticular materials can be grouped as synthetic porous solids, which result from the assembly of molecular building blocks that are connected by strong bonds to build periodic structures. These materials encompass several types, such as metal‐organic frameworks (MOFs), covalent organic frameworks (COFs), and their derivatives. Owing to the wide array of building blocks and combinations therein, reticular materials offer a myriad possibilities both in terms of structural properties and functionalities. Research in this domain gathered pace in the 1990s with the development of MOFs and was further propelled by the ability to exploit their porous properties.^[^ ^1^ ^]^ COFs, which were reported in 2005,^[^ ^2^ ^]^ benefited from prior knowledge, and this class of materials has witnessed even accelerated development. Other reticular materials, such as hydrogen‐bonded organic frameworks (HOFs), have evolved subsequently. Over the course of time, prompted by the feature to modulate functionalization chemistry, these materials have found suitability for a range of applications, such as in catalysis, drug delivery, energy storage, gas storage and separation, and water harvesting.^[^ ^3^, ^4^, ^5^ ^]^ There have been several reviews describing the different aspects of design, properties, functions, and applications of reticular materials.^[^ ^6^, ^7^, ^8^, ^9^, ^10^ ^]^ To have a holistic view of tracking this evolution over the last three decades, the current review aims to look back on the growth in the field of reticular materials by showcasing the key milestones and statistics (**Figure** **1**). In addition, the contemporary trends are presented, and the prospects and challenges that lie ahead are outlined. The discussion for each section is guided by insights from a global, crowdsourced survey of 228 researchers conducted through an online questionnaire, which was run over six months in 2024 (March–August 2024, details in the Methods section). The survey dataset not only presents a unique opportunity to condense the views of the community, but also helps measure the span and status quo of active research in reticular materials. The demographic analysis is introduced initially, followed by the advancements in standardizing and deposition of data in databases, and concentrating on academic and industrial milestones in the following sections.

## 2 Insights into the Field of Reticular Materials

### 2.1 Demographics of the Field

**Figure 2:**
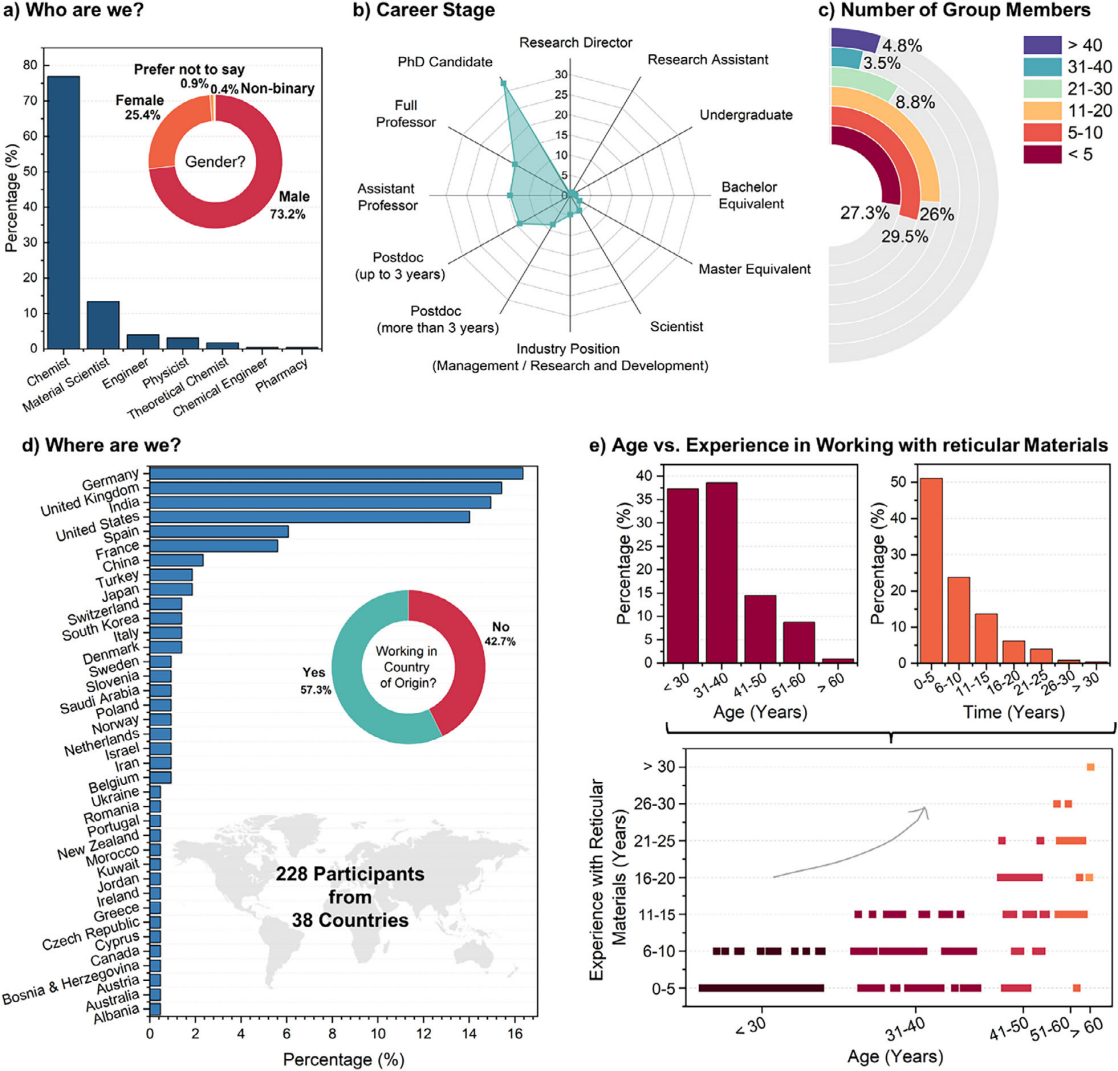
Demographics based on the survey: a) Research background and gender identification; b) career stage; c) number of group members; d) origin of researcher; and e) correlation of age of researchers vs. their experience.

The field of reticular materials has evolved from the foundations of coordination and polymer chemistry, which is reflected by the predominance of chemists (≈77%) and material scientists (>13%) as the leading cohort of expertise (**Figure** **2**a). The gradual progress and rising interest in this domain are evident in the career stage and experience of active researchers (Figure 2b,e). Young and early career researchers currently dominate the work in this space, with nearly one‐third of the respondents being PhD candidates and two‐thirds under the age of 40 (Figure 2b,e, respectively). Especially, the cross‐correlation between the age of researchers and their experience with reticular materials in Figure 2e shows a clear trend: apart from being a field attracting young minds, the research spans across the globe, with survey participants from 38 countries (Figure 2d). In 2022, an initiative called ‘MOF Universe’ was launched that creates and maintains a world map of MOF research groups.^[^ ^11^ ^]^ Although the scope is widening, research activities are not spread evenly across the globe. Likewise, there is much room for improvement to achieve gender parity, which currently is skewed (Figure 2a). Despite these concerns, a key driver for the rapid growth in this space has been the mobility of researchers. More than half of the respondents have moved out of the country of their origin (Figure 2d), enabling diversity, collaborations, and concerted efforts from larger research groups (Figure 2c).

### 2.2 Research Focus

**Figure 3:**
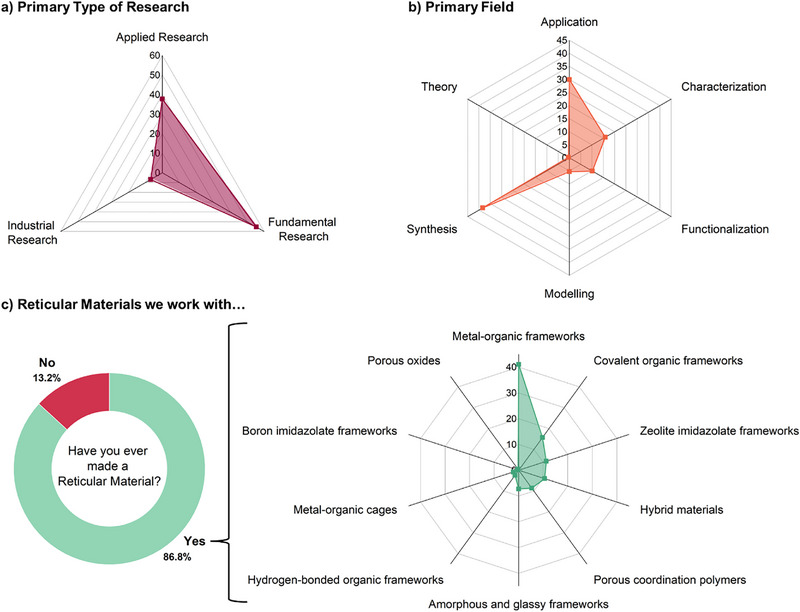
Overview of a) primary type of research, b) primary field, and c) type of reticular material we work with.

Within the field of reticular materials, the majority of >55% of research is still focused on the fundamental level, ≈38% are working in applied and only <7% in industrial research (**Figure** **3**a). Overall, the primary field of research is based on synthesis (≈38%). Application‐focused research is the second most pursued avenue in the field, while functionalization and characterization that complement the above topics are primary topics of investigation for 15% and 10% of participants, respectively (Figure 3b). The advancement of reticular materials has benefited significantly from computational and theoretical chemistry, and this number is likely to only grow with the advent of machine learning and artificial intelligence (Figure 3b).^[^ ^2^, ^6^, ^7^, ^12^ ^]^ Within the ≈87% of people stating that they have ever made a reticular material, MOFs are the preferred choice of material for research, followed by COFs (Figure 3c). However, emerging material types, such as porous liquids and derived materials, such as glassy frameworks, are increasingly commanding attention.

### 2.3 Synthesis and Characterization of Reticular Materials

**Figure 4:**
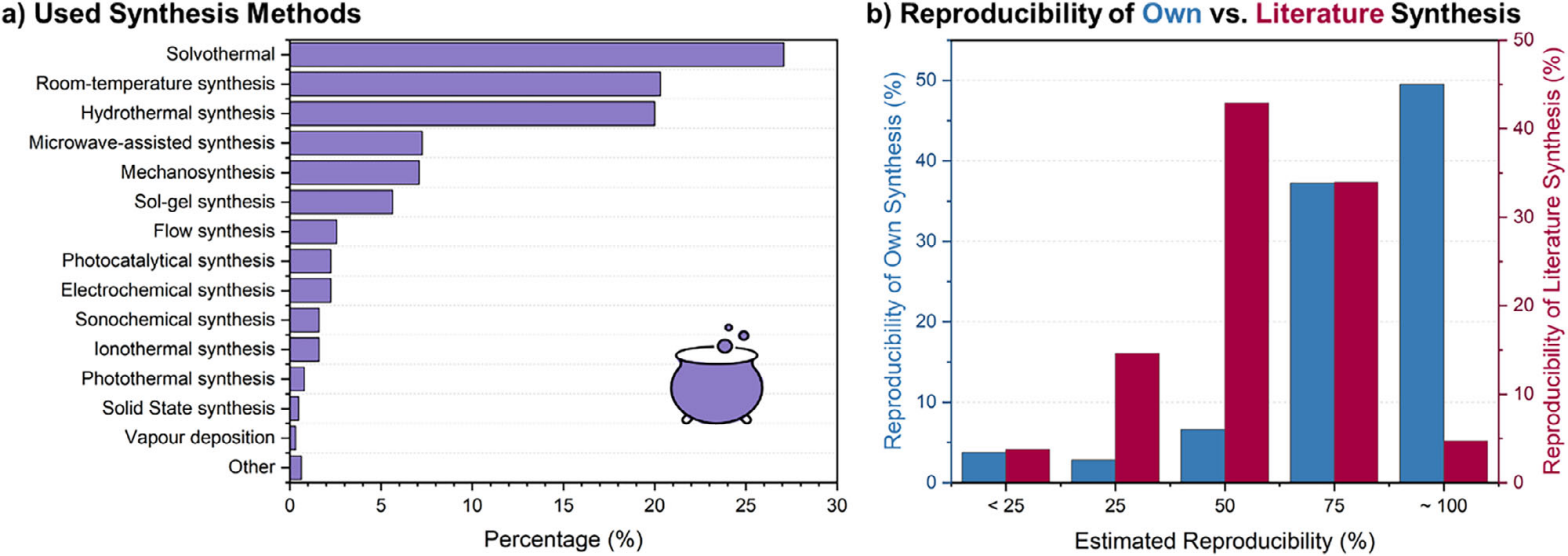
a) Overview of the percentage of the different synthesis methods and b) a comparison of the estimated reproducibility of own vs. literature synthesis.

**Figure 5:**
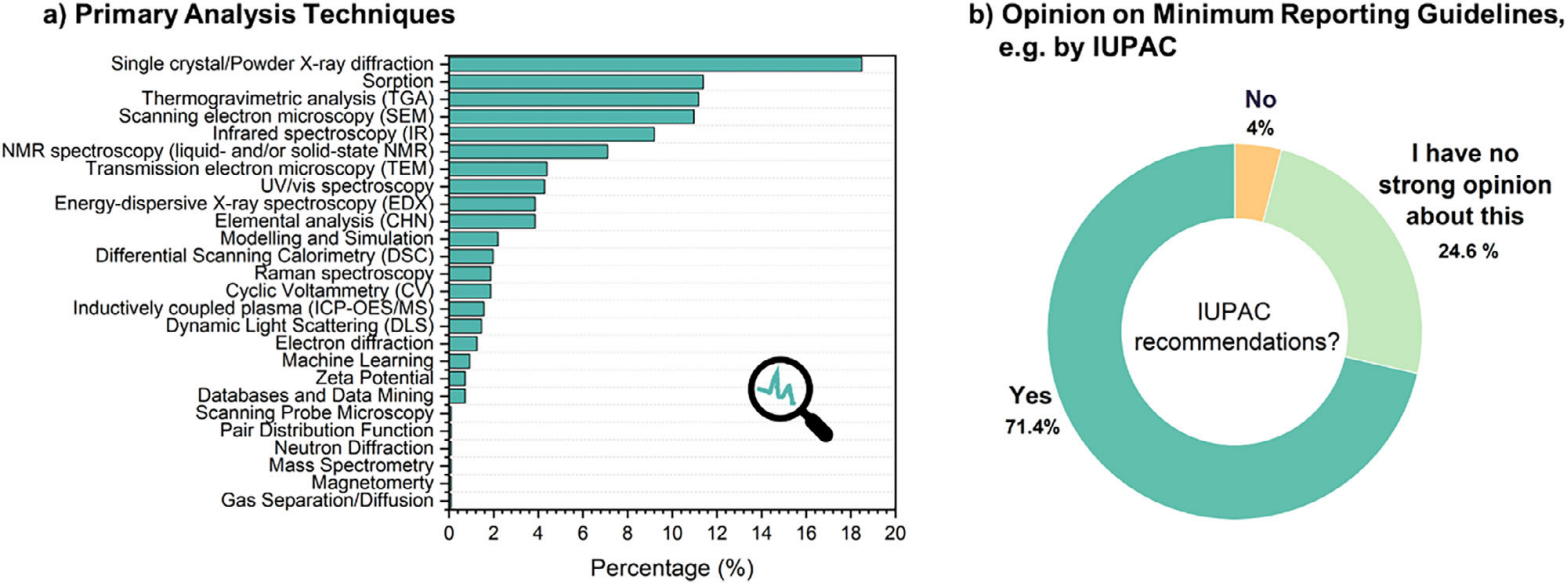
a) Primary analysis techniques and b) opinions on minimum reporting guidelines.

Reticular materials can be synthesized using several methods,^[^ ^13^, ^14^ ^]^ including, but not limited to, solvo‐/hydro‐thermal, sonochemical, microwave‐assisted, mechanochemical, ionothermal, sol‐gel, and electrochemical. According to the survey (**Figure** **4**a), the dominating method is still solvothermal (27.1%) but with a pronounced shift towards room‐temperature (20.3%) and hydrothermal synthesis (20%). The pronounced fraction is also presented by microwave‐assisted (7.3%), mechano‐ (7.1%), and sol‐gel synthesis (5.6%), while other methods, including electrochemical synthesis, are still at their emerging state. Suitability of newer techniques is being examined, and there is growing emphasis on using greener and scalable synthetic approaches as reticular materials seek commercial prospects (Figure 4a).^[^ ^15^, ^16^, ^17^, ^18^ ^]^ In line with this, synthesis‐based research continues to be the primary field for the majority of researchers (Figure 3b). In this context, reproducibility has been flagged as a growing concern across scientific disciplines, especially in chemistry research (Figure 9b).^[^ ^19^, ^20^ ^]^ Most of the respondents in the current survey have synthesized a reticular material (Figure 3c), and nearly one in two believe almost all their syntheses are reproducible (Figure 4b). This perception, however, changes significantly when thinking about reproducing known syntheses, where most respondents (≈43%) believed that only 50% of the time could they obtain the target materials using procedures in the literature (Figure 4b). In a previous survey for synthetic chemistry,^[^ ^21^ ^]^ selective reporting was suggested as one of the major factors responsible for irreproducible research, and having an experimental checklist was proposed as a way to tackle this issue. Other fields in material sciences are gradually adopting these practices, with journals requiring minimal information, such as for research in batteries, solar cells, and catalysis.^[^ ^22^, ^23^, ^24^, ^25^, ^26^, ^27^, ^28^ ^]^ Two‐thirds of the respondents in the current survey endorse this view of having a list of required information when publishing research on reticular materials (**Figure** **5**b).

Out of the more than 25 different analytical techniques for the characterization if reticular materials (Figure 5a), to date, the five most typically applied were ranked as X‐ray diffraction (SCXRD/PXRD, ≈18.5%), followed by sorption analysis (≈11%), thermogravimetric analysis (TGA, ≈11%), scanning electron microscopy (SEM, ≈11%), and infrared spectroscopy (IR, 9%). Shedding light on structural integrity as well as material composition, these five techniques complement each other perfectly, and would be a start for an initial suggestion of minimal information needed to publish new reticular materials.

### 2.4 Disseminating Results

**Figure 6:**
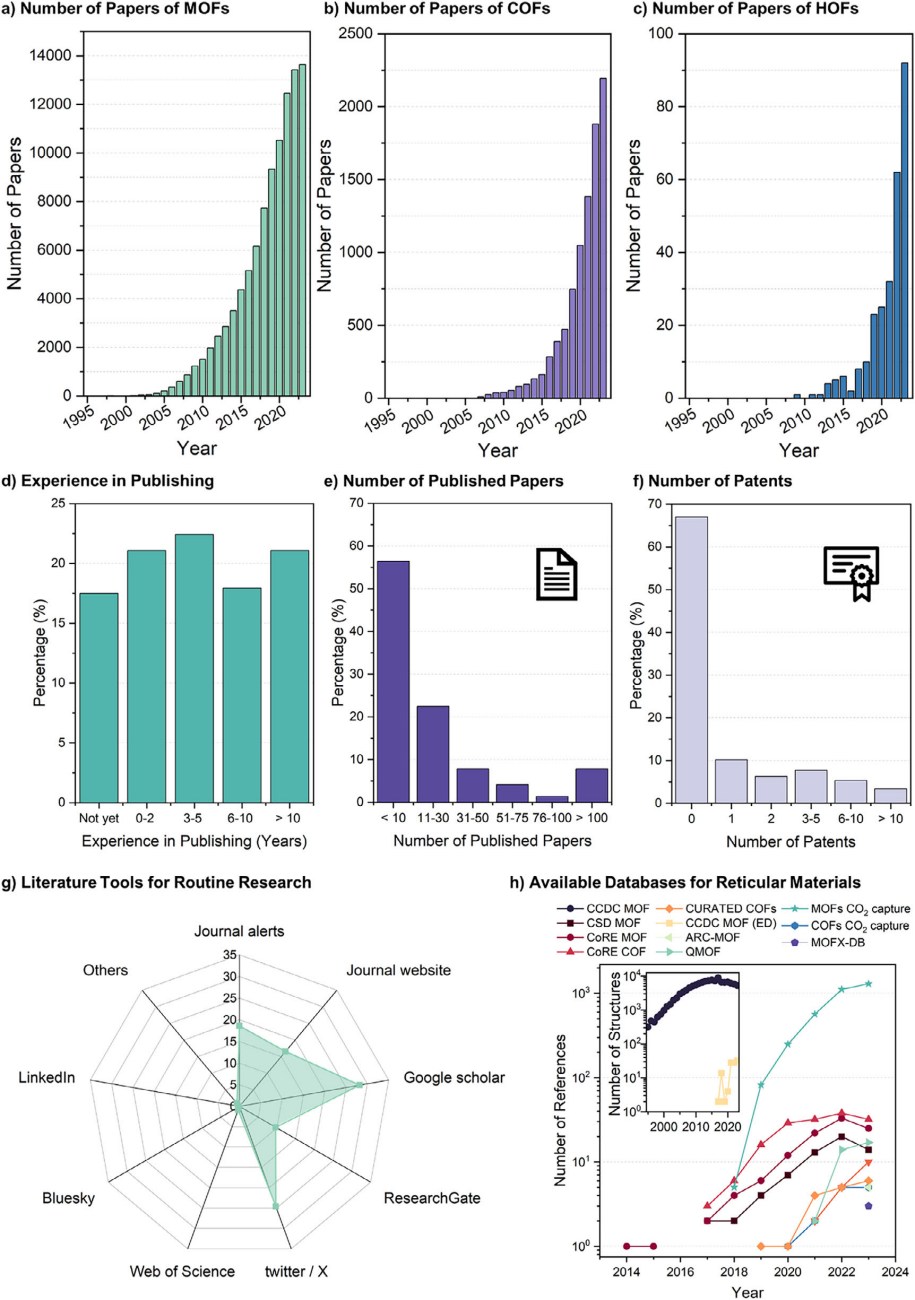
Overview of a) published MOF structures; b) published COF structures; c) published HOF structures; d) years of experience in publishing; e) number of published papers; f) number of patents; g) literature tools used for routine research and h) available databases for reticular materials.

Once synthesized and fully characterized, the resulting data is often ready for publication. The number of publications on MOFs [or porous coordination polymers (PCPs)] has grown rapidly over the last few years, with more than 10 000 publications per year for the last 4 years (2020–2023) (**Figure** **6**a). The majority of the publications are research articles (ca. 88%), but this area has also been heavily reviewed with nearly 8 900 review articles in the said period and 11 books published. Researchers and institutions from China have contributed majorly to all the publications with a 58% share. A similar trend is observed for publications involving COFs with more than 2 100 publications in 2023, and more than 1 390 review articles since this class of materials was reported in 2005 (Figure 6b). Publications based on HOFs are witnessing a gradual rise too, since early examples in the late 2000s (Figure 6c). According to the survey, there is a rather equally balanced experience in publishing between 0 to >10 years across most of the respondents (Figure 6d). Though when identifying the individual number of published papers, over 55% only published <10 papers and less than 10% more than 75 papers. This agrees with the large percentage of young researchers being in their early career stage and having <10 years of experience with reticular materials. Similarly, two‐thirds of the participants have no patents and only ≈3% have more than 10 patents (Figure 6f). With this high frequency of new published results, it becomes more and more difficult to know the most recent state‐of‐the‐art achievements. For their daily research routine, scientists mostly use Google Scholar (≈28%), followed by the social media platform Twitter/X (≈25%) and journal alerts (19%) and websites (17%), and ResearchGate (9%, Figure 6g). Nonetheless, the volume of published data is overwhelming, which raises the question of whether we should rethink our publishing behaviour to publish more impactful data rather than just more data.^[^ ^29^ ^]^

To capture the vast diversity of MOF and COF structures, researchers have produced databases to share this information with the community. There is an emerging trend in materials science to share data in online data repositories so they become accessible and usable.^[^ ^30^ ^]^

### 2.5 Use of Repositories

The Cambridge Structural Database (CSD) is the repository for small‐molecule crystal structures, which includes data for reticular materials, especially MOFs.^[^ ^31^, ^32^ ^]^ Over the last 30 years, the number of crystal structures identified as MOFs (CSD‐MOF subset)^[^ ^33^ ^]^ has risen to a total of ca. 122 900 structures (Figure 6h). The rising trend had its peak in the year 2017, after which the number of structures reported per year has reduced gradually (inset in Figure 6h). Although SCXRD has been the major technique for structure determination over the course of the last few decades, the use of 3D Electron Diffraction (ED) has seen a steady growth in the last few years (inset in Figure 6h). This technique is particularly promising for materials where growing single crystals (suitable in size for SCXRD) can be challenging, such as for COFs,^[^ ^34^, ^35^, ^36^ ^]^ HOFs,^[^ ^37^ ^]^ and many MOFs, which is likely to have a greater impact in the development of structure‐property correlations in reticular materials.^[^ ^38^, ^39^ ^]^ The number of MOF/COF databases has grown from a handful to more than ten, with many containing more than 10 000 structures. A summary of these databases is displayed in **Table** **1**, detailing the year reported, the number of structures, and whether the database contains experimental or hypothetical structures.

**Table 1:** Table of MOF/COF databases.

Name	Year	Hypothetical/Experimental	Structures	Refs.
CSD MOF Subset	2017	experimental	88 000	[33]
CoRE MOF	2014/2019	experimental	14 000	[40, 41]
CoRE COF	2017	experimental	187	[42]
Pyrene‐based MOFs	2019	experimental	62	[43]
CURATED COFs	2019/2020	experimental	648	[44, 45]
ARC‐MOF	2023	hypothetical/experimental	280 000	[46]
QMOF	2021/2022	hypothetical/experimental	20 375	[47, 48]
MOFs for carbon capture	2019	hypothetical	324 426	[49]
COFs for carbon capture	2020	hypothetical	69 000	[50]
MOFX‐DB	2023	hypothetical	160 000	[51]
ToBaCCo	2017/2019	hypothetical	∞	[52, 53]
MOF+	2016	hypothetical	∞	[54]

One of the first MOF databases was the CoRE‐MOF database, which represents a subset of the CSD that was refined in several ways, for example, to remove disorder or any guest molecules present in the pores.^[^ ^41^ ^]^ This approach was further refined,^[^ ^40^ ^]^ and the CSD has even labelled structures in their larger crystallographic database (CSD MOF subset).^[^ ^55^ ^]^ A similar computational‐ready refinement methodology was also used to create a COF dataset.^[^ ^42^ ^]^ The CoRE MOF and COF datasets are built upon reported crystal structures that classify these as “experimental” structures. However, the building block approach of these framework structures means there are also countless ways to, at least hypothetically, generate structures. Several hypothetical databases were generated to explore these structures for applications such as carbon capture.^[^ ^49^, ^50^ ^]^ Moreover, the tools to generate hypothetical databases, such as the ToBaCCo or Weaver,^[^ ^52^, ^53^, ^56^ ^]^ represent seemingly infinite structures requiring online services to explore these new structures.^[^ ^54^ ^]^ To quantify the number of references to these databases in scientific literature, we parsed the text of articles found using the Dimensions database^[^ ^57^ ^]^ that contain the citation keywords (Table 1), which represent parts of the DOIs for the publication of the respective database. We constrained our search by excluding “patents”, “grants”, and “policy documents”, while including “articles”, “chapters”, “edited book”, “proceedings”, “monograph”, and “preprints”. Since their inception, these databases have collected interest in the community with an explosion in recorded citations since 2017, Figure 6h. The outstanding number of citations for the reports demonstrates that these data are now a cornerstone for research. The data repository addresses, which represent actual data and not the manuscript, have much fewer citations (Table 1). This obfuscates our understanding of how these datasets are being used in the community, as we discussed previously.^[^ ^30^ ^]^ The next generation of databases builds upon earlier databases, refining and improving the accuracy of these structures. For example, the QMOF database leveraged an accurate density functional theory (DFT) workflow to optimise structures from the CSD MOF subset and the CoRE MOF dataset and provide useful quantum mechanical properties.^[^ ^47^, ^48^ ^]^ Similarly, the ARC–MOF database contains thoroughly structure‐validated structures from various sources using an algorithm to ensure only chemically reasonable structures.^[^ ^46^ ^]^ These rich databases of materials have spurred advancement in the community, but as outlined by Woo and coworkers, some of these structures are nonphysical.^[^ ^46^ ^]^ Future work is necessary to refine and curate these structures to improve prediction and the understanding of property‐function relationships or synthesis. Nevertheless, the exploration of structures for many different applications using these databases has accelerated research in this area, driving reticular materials towards industrial relevance.

### 2.6 Networking Opportunities

**Figure 7:**
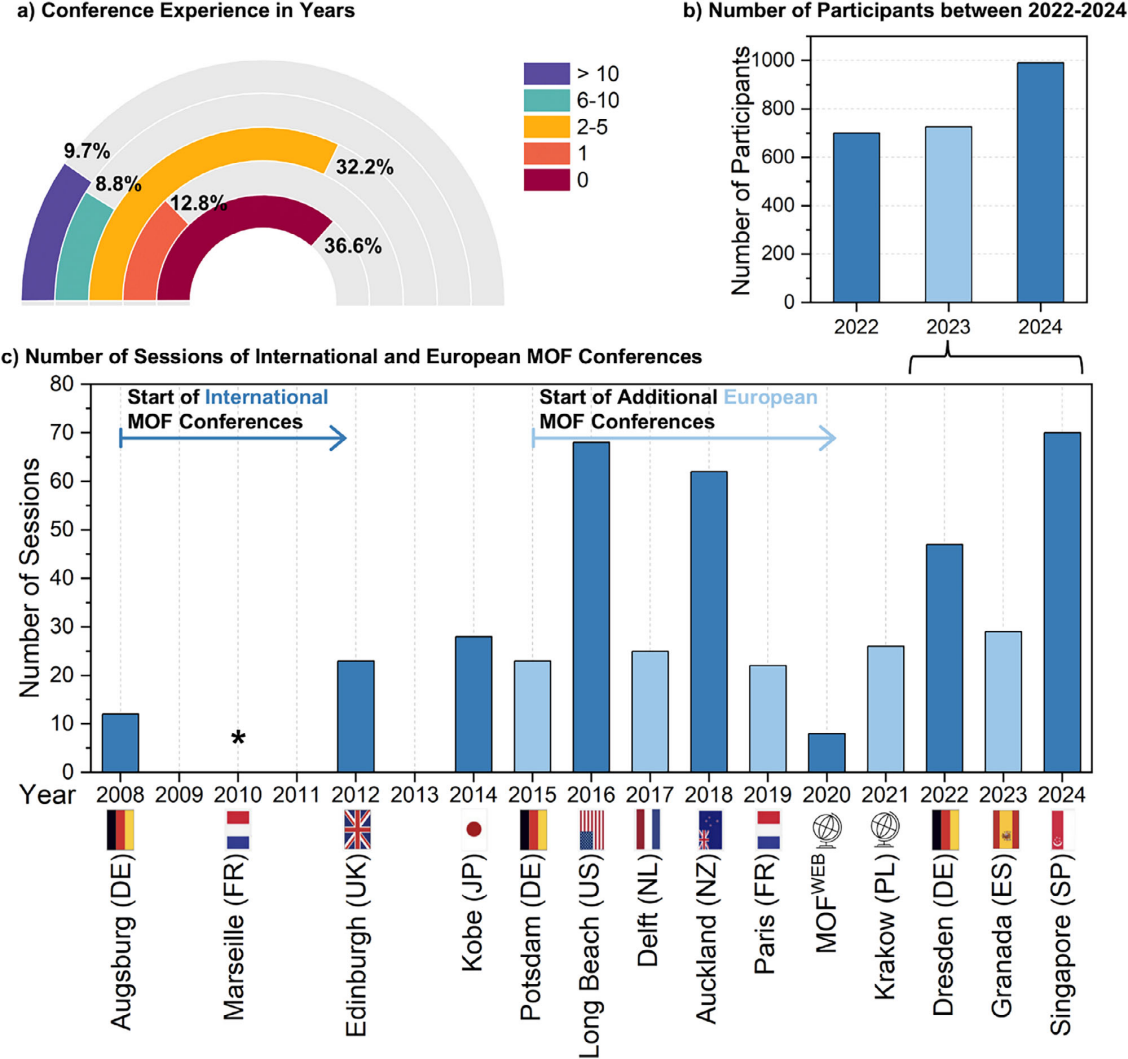
Overview of a) conference experience; b) the number of participants 2022–2024; and c) the number of sessions on a timeline of the MOF conferences; *data for the conferences without number of sessions was not available.

The mobility of researchers mentioned earlier (Figure 2d) and consequent networking in this field has further grown with the organisation of the biannual international conference, namely, International Conference on Metal–Organic Frameworks and Open Framework Compounds. This was initiated in 2008, with the first meeting held at Augsburg, Germany (**Figure** **7**c).^[^ ^58^ ^]^ Thereafter, the conference has been organised in France (2010), United Kingdom (2012), Japan (2014), United States of America (2016), New Zealand (2018), Germany (2022), with the most recent in Singapore (2024, Figure 7c). Owing to the COVID‐19 pandemic, the conferences scheduled for 2020 and 2021 were held as online events. Apart from growing from 300 participants from 24 regions in 2008,^[^ ^58^ ^]^ to >500 participants in 2016, to 630 participants in 2018,^[^ ^59^ ^]^ up to participation numbers around 1 000 from 45 regions in 2024 (Figure 7b), the widening scope of topics as well as different sessions is a testament to the increasing interest in these materials (Figure 7c).

In addition to this event other notable conferences focused on reticular materials, including (in alphabetical order) EuroMOF, French MOF, Gordon Research Conferences, MOFschools, MedPore, UK PorMat, or in association with the International Conference on Coordination Chemistry or International Zeolite Association,^[^ ^60^, ^61^ ^]^ have been organised with increasing participation and regularity. Considering the young age and career stage of the survey respondents (Figure 2e), explains the high fraction of ≈37% of people who have never been to a conference, who have been there only once (≈13%) or for up to 5 years (≈32%).

## 3 Developments in the Field of Reticular Materials

### 3.1 Academic Milestones

Most research efforts are focused on the synthesis of MOFs (Figure 3b,c); comparatively easy syntheses have gained the greatest attention. The prehistory of MOFs could be considered from 1960s with the work devoted to thermal stability^[^ ^62^ ^]^ of coordination polymers and followed by introducing a class of infinite polymer frameworks consisting of 3D‐linked rod‐like segments.^[^ ^63^ ^]^ The pioneering work of MOF‐5(Zn) synthesis^[^ ^64^ ^]^ and characterization of its crystal structure, porosity and sorption properties opened the way for a new era of tunable metal–organic networks with constant porosity and to date more than 100 000 MOFs structures with a wide range of properties are available (**Table** **2**).

**Table 2:** The extreme values of key characteristics of MOFs and COFs.

Parameter	MOF	COF
The highest surface area, m^2^/g (N_2_ BET area)	7 839 DUT‐60^[^ ^65^ ^]^	5 083 DBA‐3D‐COF‐1^[^ ^66^ ^]^
The lowest density, g/cm^3^	0.124 (H^+^‐exchange) NU‐1301^[^ ^67^ ^]^	0.106 TUS‐64^[^ ^66^ ^]^
The highest specific pore volume, cm^3/^g	5.02 DUT‐60^[^ ^65^ ^]^	5.40 COF‐108^[^ ^68^ ^]^
The smallest specific pore volume, cm^3/^g	0.03 MDC‐600^[^ ^69^ ^]^	0.14 [MeOAc‐100]_100_‐H_2_P‐COF^[^ ^70^ ^]^
The largest pore size, Å	98 IRMOF‐74‐XI^[^ ^71^ ^]^	43 JUC‐564^[^ ^72^ ^]^
The smallest pore size, Å	2.5 FMOFCu^[^ ^73^ ^]^	6 FS‐COM‐1^[^ ^74^ ^]^
The number of known structures	>100 000^[^ ^44^, ^75^ ^]^	>570^[^ ^44^ ^]^

At first, owing to exceptionally high surface area, mostly gas adsorption/storage applications of MOFs were the subject of interest, followed later by other fields including catalysis,^[^ ^76^ ^]^ biomedicine,^[^ ^77^ ^]^ and membrane technologies.^[^ ^78^ ^]^ With the advances in synthesis methods and a deeper understanding of structure crystallization processes, the variety of MOFs with specific dimensionality and properties have emerged, including stimuli‐responsive structures,^[^ ^79^ ^]^ proton‐conductive,^[^ ^80^ ^]^ magnetic,^[^ ^81^ ^]^ and 2D conjugated‐MOFs.^[^ ^82^ ^]^

With increasingly diverse application fields for reticular materials, concerns about their stability under operating conditions and greener synthesis routes for environmental and upscaling purposes arise. Since the first synthesis of MOF‐5(Zn), the combination of chemical, thermal, and mechanical stability of MOFs has been of concern,^[^ ^83^ ^]^ and within three decades it has evolved strongly. Fundamentally, the synthesis of stable MOFs is guided by strong‐bond formation between high‐valence metals (Al^3+^, Cr^3+^, Zr^4+^) with low pK_a_ ligands (e.g., carboxylic acids) as well as between divalent metals (Cu^2+^, Ni^2+^, Zn^2+^) with high‐pK_a_ ligands (e.g., azolate ligands). The formation of milestone water‐stable MOFs, MIL‐100,^[^ ^84^ ^]^ ZIF‐8,^[^ ^85^ ^]^ UiO‐66^[^ ^86^ ^]^ was followed by the development of modulated synthesis^[^ ^87^ ^]^ and post‐synthetic modification.^[^ ^88^ ^]^ Introducing rigid ligand^[^ ^89^ ^]^s, mixed‐metal nodes,^[^ ^90^ ^]^ and formation of interpenetrated structures^[^ ^91^ ^]^ also improved MOFs stability. The enhancement of MOFs stability is also assisted by computational high‐throughput screening (HTS) methods.^[^ ^92^ ^]^ In any case, the MOFs stability is connected to synthetic protocols which are now gradually evolving to satisfy green chemistry requirements. In classical synthetic routes, formamide‐based solvents are commonly used both for synthesis and washing of MOFs particles, which are hard to remove from the pore structure and are hazardous both for human health and the environment. The requirement for MOF green synthesis has given rise to several important innovative synthetic methods focused on minimizing/eliminating the use of harmful solvents, reducing energy consumption, and employing sustainable raw materials. At first, the electrochemical method was patented by BASF in 2004 (HKUST‐1),^[^ ^93^ ^]^ followed by the microwave‐assisted method in 2005 for MIL‐100,^[^ ^94^ ^]^ the mechanochemical approach in 2006 for [Cu(INA)_2_]^[^ ^95^ ^]^ and the ultrasound‐assisted (sonochemical) method in 2008 for MOF‐1 synthesis.^[^ ^96^ ^]^ The methods' evolution has been accompanied by screening for environmentally friendly solvents and reagents. The first room‐temperature water‐based synthesis was successfully demonstrated in 2015 for MIL‐53(Al) and MOF‐74,^[^ ^97^ ^]^ and attempts to replace toxic solvents with green ones are increasing up to date.^[^ ^98^, ^99^ ^]^ The weaving of green technology and life cycle assessment at each step of MOF production to develop robust scale‐up technologies will strongly accelerate MOFs adaptation to real‐world applications.^[^ ^18^ ^]^

### 3.2 Developments in Understanding Reticular Materials

The inception and development of reticular chemistry coincided with some of the most exciting decades for the development of analytical methods and instrumentation. Modern electron crystallography and low‐dose electron microscopy (EM) are techniques whose applications on MOFs, COFs, etc. are becoming increasingly accessible, despite in the early ‘90s, very little was realized or widely known about them. As soon as the research on new framework materials acquired more attention and resources, the evolution of techniques for structural and chemical analysis, as well as for the study of other numerous properties of interest, pushed the field considerably further. Not only have common characterization methods become more accurate and precise, but new insights have brought along additional structural aspects of MOFs that were never looked at before, such as flexibility, morphology, defects, and many others. This section analyzes how the research questions regarding MOF structures evolved over the past decades, stimulated by steady advances in analytical technologies. Long before MOFs were assigned their name and classification in the materials ecosystem, coordination networks were already reported, some of which contained organic molecules as linking units between metal nodes. Early examples date back to at least the late 1950's,^[^ ^100^ ^]^ when inorganic chemists already explored the fundamental question of which architecture could result from a specific combination of given metals and molecules containing binding sites, and soon formulated the idea that an enormous range of structures could be formed by combining various geometries of molecular building blocks.^[^ ^63^ ^]^ Porosity, a key identity trait of MOFs was not yet central, as the motivation and resolution of research efforts revolved primarily around the coordinative network observed in crystal structures. Crystallography, specifically SCXRD, was naturally the most relevant technique, although remarkably different and demanding compared to its modern version. As the parallel with zeolites came to the attention, research trends shifted towards obtaining metal–organic nets capable of host‐guest behaviour, projecting them towards promising real‐world applications.^[^ ^101^, ^102^ ^]^ Initially focusing on guest‐exchange processes in the liquid phase, nuclear magnetic resonance (NMR) spectroscopy and SCXRD were techniques of choice to assess host‐guest interactions at the MOF internal and external surface.

**Figure 8:**
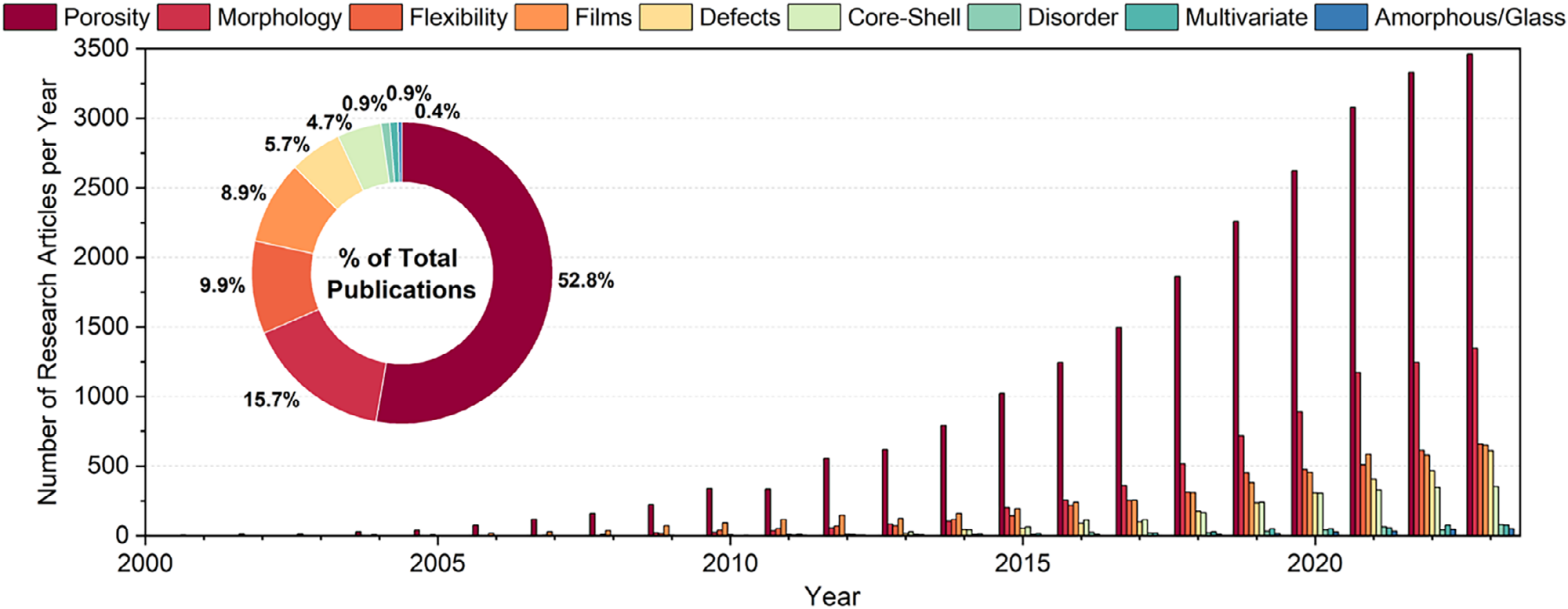
Development of ‘reticular analytics’ over time, including a percentage of the total publications.

With the discovery of MOFs maintaining their porosity upon evacuation, ‘permanent porosity’ became a leading research topic, and gas‐sorption measurements (particularly N_2_ adsorption) stably entered the characterization checklist of so‐called ‘reticular’ chemists (**Figure** **8**).^[^ ^103^, ^104^ ^]^ Focusing on the internal surface of MOFs, a natural consequence of an attention shift towards changing adsorption behaviour was producing structures with different molecules to afford larger pore spaces, a specific surface chemistry, or more extended internal surfaces.^[^ ^105^, ^106^ ^]^ However, crystal morphology can also drastically impact sorption properties, particularly when the exterior surface—readily available to the crystal surroundings compared to the inner cavities—is a relevant fraction of the overall surface.^[^ ^107^ ^]^ The study of MOF morphologies was arguably ignited by gas‐sorption studies, but it has not been limited to gas‐sorption or separation behaviour. To gain insight to the development of the “reticular analytics” over the course of the past 30 years nine different categories were evaluated with a SciFinder keyword search (details see chapter 7): porosity, morphology, flexibility, films, defects, core‐shell, disorder, multivariate and amorphous/glass (sequence with decreasing total amount, Figure 8). Up to 2000, there was only one research article published per year focusing on porosity, but this number then grew rapidly to 40 articles in 2005, 340 articles in 2010, over 1000 articles in 2015, and almost 3500 articles in 2023. In total, the articles published with a focus on porosity sum up to more than 50% of all publications. Over the years, the diversity of ‘reticular analytics’ grew, and besides porosity, the aspects of morphology (15.7%), flexibility (9.9%), thin films (8.9%), as well as defects (5.7%) and core‐shell composites (4.7%) became more and more relevant. Further research aspects such as defects (0.9%), multivariate (0.9%), and amorphous MOFs (0.4%) gain increasing attention.

Tuning MOF structures into nanoparticles,^[^ ^108^ ^]^ core‐shell composites,^[^ ^109^ ^]^ thin films,^[^ ^110^ ^]^ and nanowires^[^ ^111^ ^]^ became a distinct research trend in numerous other applied fields of chemistry, such as catalysis, medicine, and even electronics, textiles, and metamaterials. From an analytical point of view, this research area came with the adoption of imaging techniques such as fluorescence imaging,^[^ ^112^, ^113^ ^]^ multimodal optical spectroscopy and in situ imaging correlating (MOSAIC) approach,^[^ ^114^, ^115^ ^]^ Scanning and Transmission Electron Microscopy (SEM and TEM)^[^ ^116^ ^]^ and Atomic Force Microscopy (AFM), although in this context no substantial advances were required to retrieve the desired morphological information.

The study of MOFs as adsorbents highlighted another structural characteristic that became almost an additional identity trait: being ‘soft’, flexible structures.^[^ ^117^ ^]^ While the presence of organic linkers endows MOFs and COFs with higher torsional flexibility at the local scale compared to other inorganic porous compounds, the discovery of conformational ‘switchability’^[^ ^118^ ^]^ highlighted a design pathway towards a more versatile range of accessible internal volume and surface, and peculiar behaviours such as dynamic phenomena,^[^ ^119^, ^120^ ^]^ negative thermal expansion^[^ ^121^, ^122^, ^123^, ^124^, ^125^, ^126^ ^]^—also common among zeolite structures—and negative gas adsorption.^[^ ^127^ ^]^ Conformational freedom is currently widely recognized as a key aspect of MOF behaviour, and a research topic on its own, which even saw the appearance and transition through different “generations” of framework flexibility. A closely linked research topic is the study and engineering of dynamical conformational freedom, or simply “framework dynamics”,^[^ ^128^ ^]^ which has been strongly enabled by solid‐state NMR. Overall, the research interest in conformational flexibility and framework dynamics is so closely intertwined that the relative communities are mostly overlapped, and the use of both gas‐sorption and NMR‐based techniques is often paired to better understand the relationship between these aspects, and their consequences on emergent properties.

Other than assessing the effects of structure, morphology, and flexibility, the widespread practice of measuring the internal surface of MOFs also gave some of the earliest indications that average crystal structures are approximate representations of their real complexity. Indeed, deviations from the expected porosity could only be assigned to measurement/method imperfections or real‐structure features such as defects and static structural disorder.^[^ ^129^, ^130^ ^]^ Nevertheless, these were —and, to a good extent, still are—particularly elusive to characterize. Specifically, the quantity of defects and the degree of static distortion can be derived by combining gas adsorption behaviour with a range of chemical analyses, but the local distribution of these characteristics required the adoption of more recent diffraction and electron microscopy techniques such as pair‐distribution function (PDF) analysis and TEM.

While the first mention of defects in the MOF field referred to both surface imperfections due to crystal growth,^[^ ^131^ ^]^ crystallographic defects,^[^ ^132^ ^]^ only the latter type was set to become a distinct field of research, named “defect engineering”.^[^ ^133^ ^]^ Over the past decade, this field has matured considerably, and its focus has undergone significant changes. Most notably, from the earliest studies on the quantification of vacancy defects, the attention shifted towards a much greater challenge, that is, deciphering the distribution of defects—i.e. the local structure—in the crystal architecture.^[^ ^134^ ^]^ Moreover, substitutional defects started to become an exciting feature with the advent of so‐called multivariate MOFs, generally consisting of mixed‐building‐block architectures structurally analogous to solid solutions, where the mixed components occupy specific crystallographic sites, but are non‐periodically distributed in the extended framework.^[^ ^135^ ^]^ As it became clear that the distribution of vacancies and chemical species may have an unpredictably relevant influence on the host‐guest properties of MOFs and other framework compounds, conventional crystal structure determination by diffraction techniques showed its limitations as it cannot provide spatially resolved information on non‐average features. With local structures entering the spotlight of MOF synthesis, techniques capable of probing short‐ranged order went from exotic to frequently needed practices. One of these techniques is TEM. Being that it was traditionally developed on inorganic compounds, commonly withstanding orders of magnitude stronger electron beam intensities, imaging MOF structures down to atomic resolution to spot local arrangement of defects started as an ambitious goal. If on the one hand the use of TEM in biomolecular sciences provided useful indications and methods to address the extreme beam sensitivity of molecule‐based framework material, imaging‐based structure analysis of proteins and other biomolecules can be conducted on single particles by analyzing them in large numbers and merging the resulting information to obtain a 3D reconstruction of the macromolecule. This is fundamentally different from the needs of MOF chemists, who need to analyze crystals and cannot conduct the same convenient practice of averaging projection images of thousands of specimens. For this reason, MOF samples became increasingly common as test systems for the development of TEM techniques best suited for particularly beam‐sensitive compounds, such as Differential Phase Contrast‐STEM (DPC‐STEM),^[^ ^136^ ^]^ Contrast Transfer Function‐corrected TEM,^[^ ^134^ ^]^ or electron ptychography.^[^ ^137^ ^]^ Complementary to imaging, powder X‐ray scattering techniques focusing on diffuse scattering gained more attention as a non‐destructive tool to investigate local structures from a much more representative fraction of the sample (at least thousands of crystals) compared to the information achievable by TEM, which is highly precise but not necessarily accurate. Short‐range order can indeed be obtained from powder XRD measurements by converting diffraction data into a PDF, which represents a histogram of all the atom‐atom contacts in the material. This technique, established decades before the inception of reticular chemistry,^[^ ^138^ ^]^ became increasingly valuable not only for analyzing defects,^[^ ^139^ ^]^ but also for investigating ambiguous average structures,^[^ ^140^, ^141^, ^142^ ^]^ giving insight into solution state synthesis,^[^ ^143^, ^144^ ^]^ and even for structure determination of amorphous frameworks or nanoparticles.^[^ ^145^, ^146^, ^147^ ^]^ Indeed, the widespread availability of suitable X‐ray instrumentation and freeware for PDF analysis surely had a critical role in promoting the beginning of a still relatively small area of MOF research dedicated to the development and study of glassy MOFs and their unique properties.

While PDF analysis from PXRD data showed its invaluable role in local structure analysis, it is, however, not ideal for elucidating the distribution of defects across the extended structure. The reason is that, as it relied on 1D data, this technique is insensitive to crystallographic directions and provides radially averaged interatomic distances, whose overlap becomes problematic already at a range of a few unit cells, resulting in an overall homogeneous background with no retrievable structural information. Recently emerging alternatives that provide 3D‐resolved local structure information while being complementary to TEM imaging are atom probe tomography^[^ ^148^ ^]^ and 3D total scattering analysis^[^ ^142^, ^149^ ^]^—both conducted on single crystal specimens. Although still rare and poorly accessible for the global MOF and COF community, a few case studies showed their great promise for future more widespread use, promoted by the establishment of standardized practices and user‐friendly software for data processing and aperiodic structure determination.

Another technique that certainly deserves mention is electron crystallography. Started as a relatively niche kind of use in TEM microscopy, it underwent numerous changes in “flavor” over time as various data acquisition methods and names for the technique itself emerged over the past two decades. Nowadays, electron crystallography has become mostly known as 3D electron diffraction (3D ED),^[^ ^150^ ^]^ and its data acquisition methodology is generally settled as reminiscent of conventional SCXRD, i.e., continuous sample rotation with simultaneous acquisition of diffraction images. On the one hand, the entrance of 3D ED in the field of MOFs has not yet created new research directions in the field; on the other hand, it has made structural analysis more accessible and accurate when samples for SCXRD are not available. In fact, not only structures that were too complex to solve by PXRD were elucidated by ED,^[^ ^151^, ^152^, ^153^ ^]^ are also becoming increasingly common as the availability of this technique spreads in the reticular chemistry community. This trend is projected to accelerate also thanks to the emergence of the first commercial electron diffractometers,^[^ ^154^ ^]^ with notable consequences on the very synthetic chemistry of MOFs, such as, for example, the promotion of more environmentally friendly mechanochemical synthesis routes. In perspective, the transition of 3D ED as well as SCXRD to becoming 3D total scattering techniques is expected to expand the structural precision of modern crystallography considerably further, and to bring along more sophisticated questions and research directions in our field.

### 3.3 Industrial Milestones

Breakthroughs in material science have often been made possible through effective collaborations between academic researchers and industry professionals. For example, the development of industrial‐scale catalytic processes was significantly accelerated by such partnerships. Especially, the relatively young field of reticular chemistry is now enjoying a growing contribution of industrial start‐up companies that have been founded by academic researchers and pioneers in the field. To date, industrial commercialization of MOFs includes both the scale‐up synthesis optimization, processability enhancement, and introducing MOFs into industrial cycles (such as gas storage, CO_2_ capture, water harvesting). Despite that >100 000 MOF structures have already been synthesized, only a few of them are finding their place in industrial and daily life applications. Despite trends towards greener/cheaper synthesis routes, which would be more attractive for upscaling (Figure 4a), laboratory‐synthesized MOFs published in the literature could even have outstanding characteristics, for instance, the highest surface area, but they are hard to scale up.

**Figure 9:**
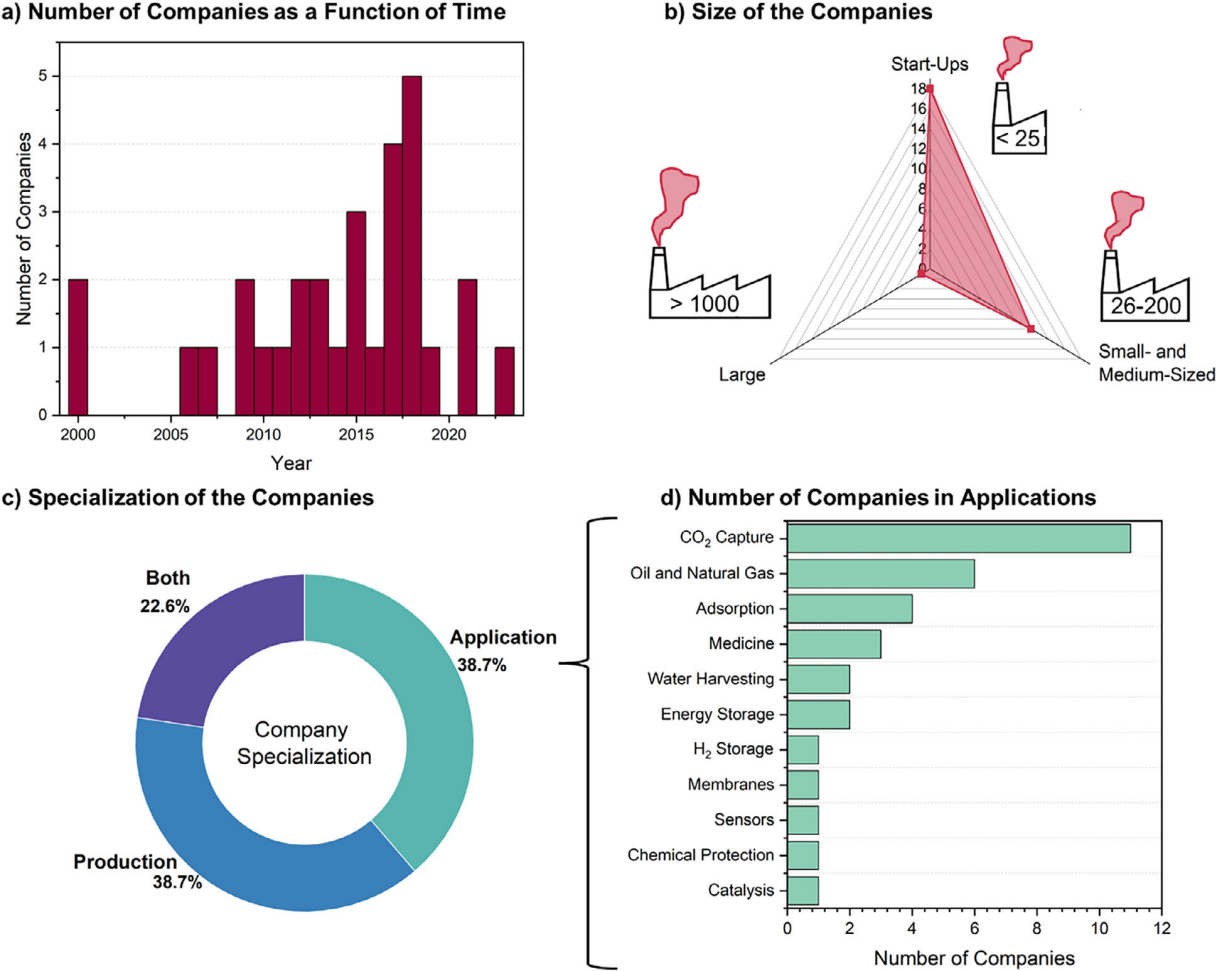
Industrial Milestones: a) number of companies with respect to time; b) size of the companies; c) specialization of the companies; and d) number of companies in the different applications.

To track the dynamics of MOFs' commercialization, a timeline of MOF companies, their company size (large, medium, start‐up), and their specialization were evaluated (**Figure** **9**).

At early stages of MOFs development, the commercialization of MOFs was supported by BASF, the first company that created a pathway for the commercial adaptation of MOFs, both focusing on synthesis scale‐up and real‐world applications; and to date, successfully led their production. Considerable efforts in this regard are also made by NuMAT, which is equipped to upscale and tailor MOFs to targeted applications. The other mature company, Strem Chemicals UK Ltd., is specializing in the production and distribution of MOFs materials and ligands for MOFs synthesis.

Starting from 2006, one by one, companies dedicated specifically to MOFs started to be established. The period 2015–2018 can be regarded as “a blooming period” with a maximum of 4–5 companies per year (Figure 9a). The dominating type of companies to date are micro‐sized (start‐ups), which commonly take their origin in research institutions and universities (Figure 9b). The number of small and medium‐sized companies is promising, and there is always a tendency for start‐ups to grow to a larger‐size companies. The number of companies involved in the production and application of MOFs is equally distributed (Figure 9c). The key leading industrial areas are CO_2_ capture and oil/natural gas industry, which is expected as these areas are in highest demand, and the MOFs high surface areas and tunable porosity are promising here (Figure 9d).

The other applications, including medicine and catalysis, are in their infancy and emerging states. This contrasts with the growing number of academic publications devoted to medical and catalytic MOFs studies. To date, the most representative commercially available MOFs are Basolite A520 (BASF), CALF‐20, SENTINEL‐110 for natural gas storage, CO_2_ capture, and chemical protection, respectively (**Table** **3**). To expand this list, there should be a complementary effort taking both from academic laboratories and industrial companies to achieve a sustainable level of MOFs commercialization.

**Table 3:** Distribution of MOFs types between application fields. Representative MOFs are being commercialized.

Industrial field	MOFs type
Natural gas storage	Basolite A520 (BASF) SA = 1300 m^2^ g^−1^ (N_2_ BET)^[^ ^155^ ^]^ uptake 139.2 (140) mg/g (200 bar, 303K, volumetric)^[^ ^156^ ^]^
CO_2_ capture	CALF‐20^[^ ^157^ ^]^ (Svante and BASF) SA = 528 m^2^ g^−1^ (Langmuir), 38% void volume, Selective CO_2_ physisorption: CO_2_ uptake 4.07 mmol g^−1^ at 1.2 bar and 293 K. Capture up to 95% of CO_2_ emitted from industrial sources (cement, blue hydrogen plants).
Chemical protection	SENTINEL‐110 (NuMat) SA = 1000 m^2^ g^−1^; Nitric oxide (NO) gas breakthrough is 66 min

Considering the industrialization of other reticular materials, only a few companies are known. For COFs commercialization, the first company YOCOF MATERIAL CO. LTD was founded in 2023, focusing on the production of COFs sorbents. For commercialization of porous liquids, Porous Liquids Technologies company was founded in 2015, specializing in the production of porous liquid technologies for CO_2_ and noble gas capture and hydrocarbon separation.

## 4 Challenges for Reticular Materials

**Figure 10:**
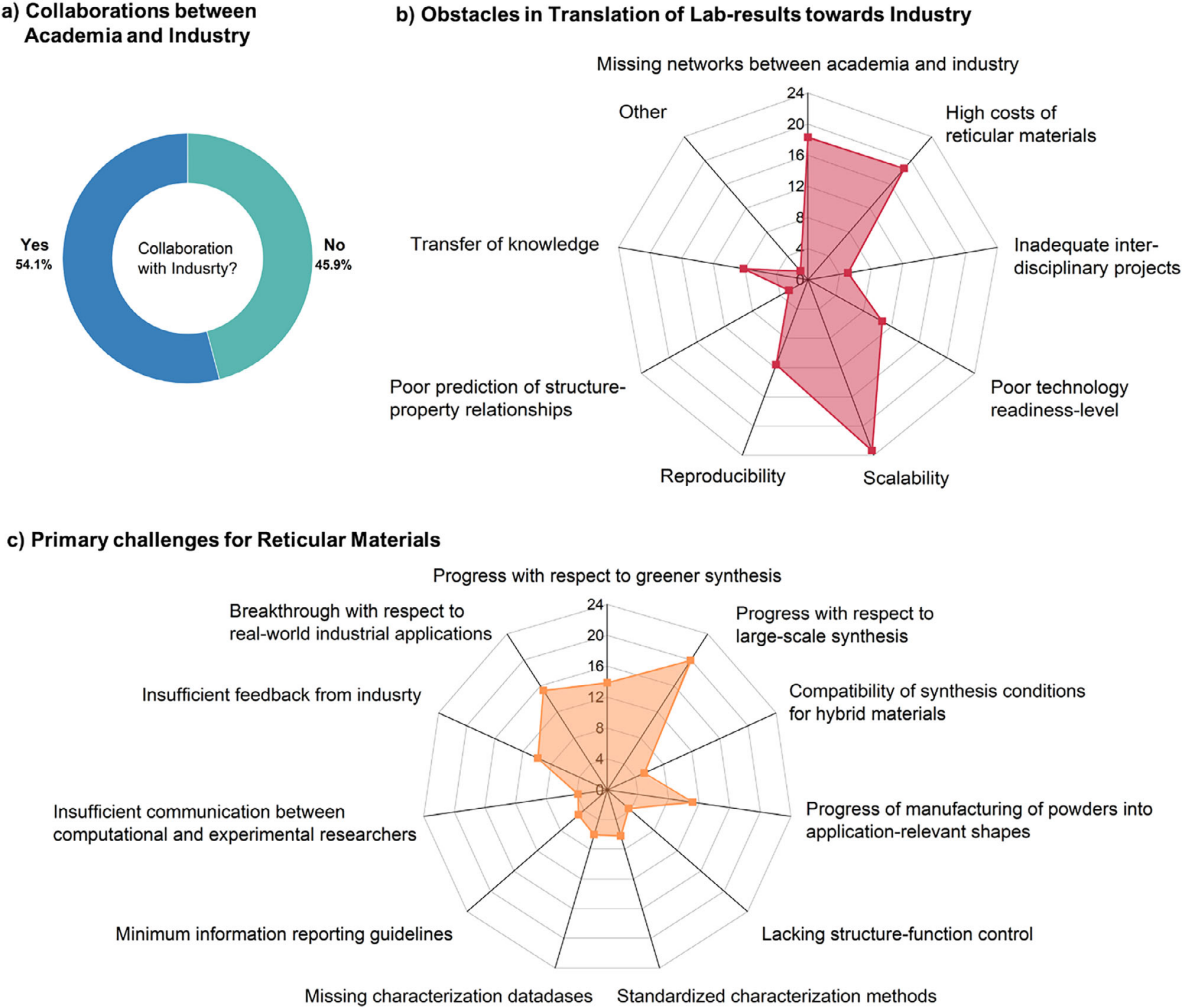
a) Involvement in collaborations between academia and industry, b) hindrances for the translation of lab results to industry, and c) primary challenges for reticular materials.

The current landscape of reticular materials necessitates strong industry‐academia ties to facilitate the transition of lab‐scale innovations to commercial products. The fact that slightly more than half of the respondents (54.1%) are engaged in collaborations between academia and industry shows progress, but also highlights the potential for expanding these partnerships further (Figure **10**a).^[^ ^158^ ^]^ Bridging the gap between the two sectors can enhance the scalability, practicality, and economic viability of new materials as a direct exchange may guide fundamental research and industrial goals alike. Encouraging more widespread and systematic collaborations can help address the remaining challenges. For instance, initiatives like joint research projects, industry‐funded academic programs, and collaborative grant opportunities can be promoted to enhance this trend and further advance the field of reticular materials towards real‐world applications.

The key hindering factors for the translation of lab results to industry in Figure 10b are scalability (23.3%), high costs of reticular materials (18.7%), and missing networks between academia and industry (18.3%). Other notable barriers include reproducibility (11.6%) and poor technology readiness level (10.7%).^[^ ^7^ ^]^ Scalability is a fundamental challenge in translating lab‐scale findings to industrial processes. The high percentage indicates that while lab‐scale innovations are promising, their industrial application often requires significant adaptation and optimization. The difficulty may lie in the variability for certain syntheses that may be successful in a small batch (lab scale) but face entirely different challenges when the innovations need to be scaled up (industrial scale). This brings in other concerns of potentially toxic, harmful, and sheer volume of solvent waste streams that are less of a concern in a small lab scale but might be a deciding factor during the industrial scale‐up. Furthermore, the high costs associated with reticular materials (metal salts and specialized linkers) to target a specific application present a significant barrier to their widespread adoption. Reducing these costs requires advancements in synthesis methods and economies of scale.

These obstacles may be solved through innovative engineering solutions and large‐scale pilot plants, which in turn bring more financial burden with them. However, through a more in‐depth exchange between industry and academia, these challenges could be addressed during the development stage of new, innovative products, and a profitable solution might be found early on. Transparent communication on both sides may be needed to guide innovation early and ensure standardization of relevant reports that reflect real‐world application conditions, which may be translated more easily into business opportunities.

This may further be challenged by the second major hindrance that the community identified, which are missing networks between academia and industry. This underscores the need for better communication and collaboration channels. Effectively bridging this gap would present new opportunities to address the scalability and associated higher cost. Current efforts should focus on creating more structured networking platforms and collaborative frameworks.

These issues highlight the technical and evolving challenges in the field. Ensuring reproducibility and enhancing the technology readiness level are crucial for gaining industrial trust and investment. This requires rigorous standardization and validation processes, which have been crucial in fields such as pharmaceuticals, semiconductor manufacturing, and the more mature field of porous materials – zeolite materials, which are widely used in catalysis. The desire to have such minimum reporting guidelines and standards is reflected in the wish of ≈71% of the survey respondents to have IUPAC recommendations (Figure 5b).

The primary challenges identified by the respondents include progress with respect to large‐scale synthesis (19.9%), breakthroughs in real‐world industrial applications (15.2%), and greener synthesis methods (13.9%). Other challenges involve the manufacturing of powders into application‐relevant shapes (11.1%) and insufficient feedback from industry (9.9%) (Figure 10c).

The most prominent challenge is large‐scale synthesis, reflecting the difficulty in scaling up production from the lab to an industrial scale. This challenge is not unique to reticular materials but is a common hurdle in materials science. This is a significant hindrance (as discussed above) but has been previously demonstrated in the now well‐established field of zeolites, as well as the mass production of silicon wafers, that overcoming this challenge requires significant investment in process engineering and infrastructure.

The need for breakthroughs in real‐world industrial applications signifies the gap between theoretical research and practical use. The transition from theoretical potential to industrial application has been marked by key innovations that demonstrated clear benefits over existing technologies (e.g., semiconductor chips, lithium‐ion batteries, etc.). For reticular materials, identifying and optimizing specific applications where they offer distinct advantages and performances is crucial.^[^ ^17^, ^18^ ^]^ The emphasis on greener synthesis methods reflects the increasing importance of sustainability in materials science. The shift towards greener practices has often been driven by regulatory pressures and societal demand. For reticular materials, developing environmentally friendly synthesis routes can not only improve their marketability but also align with global sustainability goals. Many studies have proven that reticular materials have the potential to be synthesized in “green” ways, including mechanochemical strategies, or tweaking the synthesis protocols so that water may be used as solvent.

The challenges of manufacturing powders into application‐relevant shapes and potentially insufficient feedback from industry highlight the need for better integration of industrial requirements into academic research and vice versa, which may see improvement in the advent of multiple start‐up companies in these fields. Successful highlights the need for better integration of industrial requirements into academic research. Successful materials have often been those whose development was guided by continuous industry feedback, ensuring that the research remains aligned with practical needs. Generating a mutual understanding of what makes materials need to endure (shaping) may address this hindrance early on, paving the way for materials that prove stable even when manufactured into industry‐relevant shapes.

This comprehensive overview, including the insights of active researchers, highlights the current state and challenges that some of the reticular materials community is facing with respect to the exchange between academia and industry. The involvement in direct collaborations shows promising trends but also highlights the need for more widespread partnerships. The identified hindrances, such as scalability, high costs, and missing networks, underscore the critical areas that need to be addressed to facilitate the transition of lab‐scale innovations to industrial applications. The primary challenges, including large‐scale synthesis, real‐world applications, and greener synthesis methods, reflect the ongoing efforts needed to advance the field. Generating a mutual exchange and understanding of critical challenges between the two may help eliminate some of these highlighted hindrances. Strategic efforts to enhance collaborations, reduce costs, and focus on sustainable practices can significantly propel the field of reticular materials forward.

## 5 Prospects of Reticular Materials

**Figure 11:**
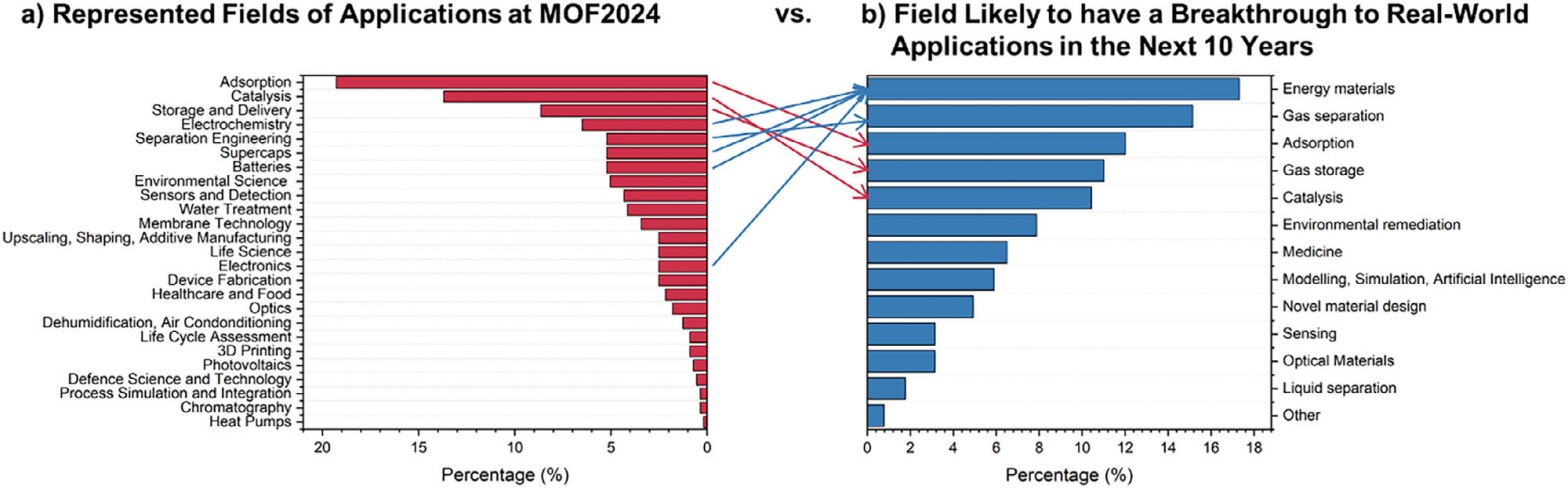
Represented fields of applications at MOF2024 in Singapore vs. b) fields likely to have a breakthrough in real‐world applications in the next 10 years.

After the evaluation of the demographics, key developments, milestones, and challenges of the field, the future prospects can be assessed. According to the survey, the field the researchers work in, interestingly, does not align with the field they think is likely to have a breakthrough in the next 10 years (**Figure** **11**a). Based on the represented research field at MOF 2024, among the areas researchers are working on, adsorption (≈19%) ranks first, followed by catalysis (≈14%) on the second, and storage and delivery (9%) on the third. However, regarding the breakthrough fields, these are only third, fifth, and fourth, respectively. Most survey respondents ranked the field of energy materials (≈17%) and gas separation (≈15%) as the most likely ones to have a breakthrough (Figure 11b).

**Figure 12:**
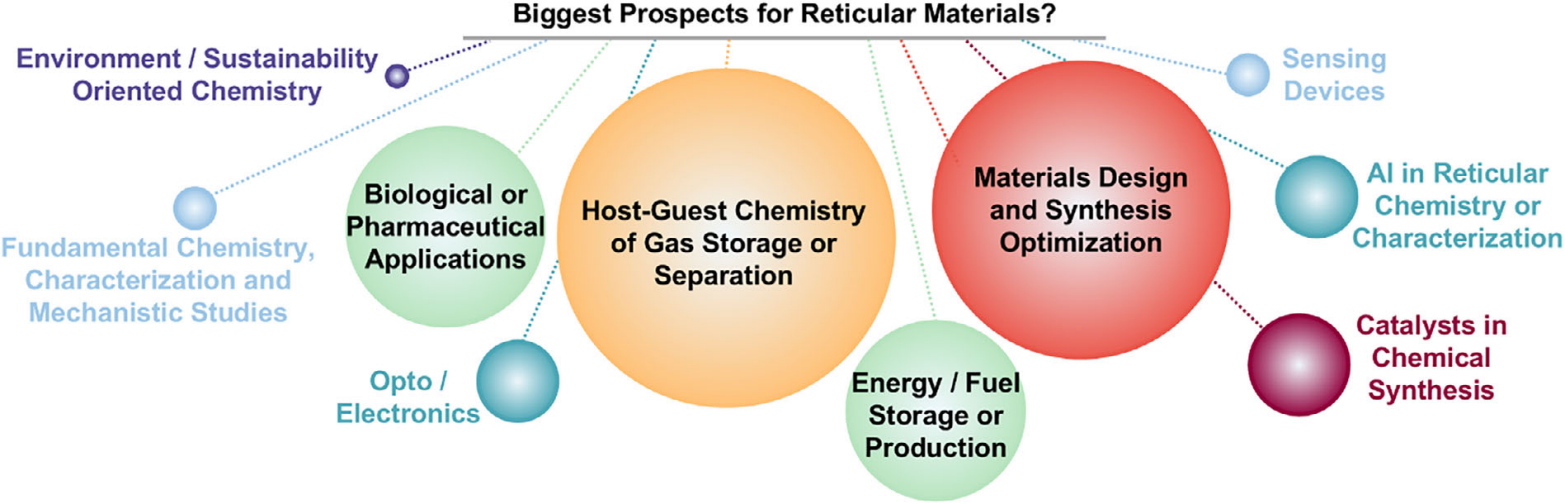
Overview of the summarized answers regarding the biggest prospects of reticular materials, with the size of the bubble indicating the relevance of the prospect.

While these fields are the ones expected to achieve breakthrough results, the biggest prospects are foreseen in terms of “Host‐Guest Chemistry of Gas Storage or Separation”, “Materials Design and Synthesis Optimization”, and “Biological or Pharmaceutical Applications” (Figure  11). More and more evolving, also the importance of artificial intelligence for reticular materials is being perceived.^[^ ^159^, ^160^ ^]^ Interestingly, in contrast to the >55% of the research that is still focused on the fundamental level, only a very small fraction sees big prospects in “Fundamental Chemistry, Characterization and Mechanistic Studies” (**Figure** **12**).

## 6 Conclusion and Outlook

This review has retrospectively traced the growth of reticular materials over the past three decades by highlighting the statistics and key milestones. Progress at multiple levels, including fundamental research and adoption by industry, has demonstrated real‐world potential and commanded attention from other disciplines of science and engineering. For instance, in their outlook for chemical innovations that could make our planet more sustainable, IUPAC listed MOFs and other porous materials as one of the top 10 candidates.^[^ ^161^ ^]^

This review article also features a first‐of‐its‐kind survey for researchers working with reticular materials. The outcomes have enabled measuring the demographic dimensions and have helped condense the opinion of the community. The collective viewpoint also allows acknowledging gaps in the field, such as issues with replicating synthesis and achieving scalability. A possible solution could be recognizing best practices,^[^ ^162^, ^163^ ^]^ identifying discrepancies through round‐robin experiments,^[^ ^164^, ^165^, ^166^ ^]^ and extensive sharing and archiving data in repositories.^[^ ^30^, ^167^ ^]^ Use of machine learning models has already exhibited immense potential in accelerating material discovery,^[^ ^48^, ^168^ ^]^ and navigating through large databases to screen materials for specific applications.^[^ ^169^, ^170^, ^171^, ^172^ ^]^ These approaches further benefit from the use of high‐throughput experimental methods and rapid characterization techniques. Apart from identifying the optimal material, the cost, poor technology readiness levels, and environmental impacts of production are some of the other major concerns raised in the survey. In this regard, developing greener synthesis routes,^[^ ^173^, ^174^ ^]^ and estimating cost,^[^ ^175^, ^176^ ^]^ and sustainability metrics^[^ ^18^, ^177^, ^178^, ^179^, ^180^ ^]^ of material synthesis are attainable targets. Through coordinated efforts between industry and academia, the transition of reticular materials from lab‐to‐market^[^ ^7^, ^181^ ^]^ may be promoted, yielding new industrial milestones and unlocking their full potential.

## 7 Experimental Section

### Survey Method

The online survey was conducted between March 2024 to August 2024. This survey received ethics approval code CH17723 (University of St Andrews). The language for the questions and responses was English, and to encourage participation from researchers at all career levels, an inclusive approach was adopted in the questionnaire. Respondents were actively invited via direct emailing, promotion on social media, and advertisement at various conferences. A total of 228 participants provided their feedback.

### Publication Analysis

To analyze the number of publications in the literature, the search on the Web of Science was employed.^[^ ^182^ ^]^ The time range was taken between 1994–2023, and the keywords for MOFs were “metal‐organic framework or metal‐organic frameworks” or “porous coordination polymer or porous coordination polymers”. Likewise, for COFs, the search term was “covalent organic framework or covalent organic frameworks”, while for HOFs, it was “hydrogen‐bonded organic framework or hydrogen‐bonded organic frameworks”. *Citation analysis*: To quantify the number of references to these databases in scientific literature, we parsed the text of articles found using the Dimensions database^[^ ^57^ ^]^ that contain the citation keywords (Table 1), which represent parts of the DOIs for the publication of the respective database. We constrained our search by excluding “patents”, “grants”, and “policy documents”, while including “articles”, “chapters”, “edited book”, “proceedings”, “monograph”, and “preprints”.

### Analysis of the Developments of the Analytical Methods

To quantify, a SciFinder keyword search was done. Herein, “metal‐organic framework” AND “*keyword*” was searched, except for amorphous/glasses, where “amorphous metal‐organic framework”, “metal organic framework glass” was searched. The same was performed with “MOF” instead of the full acronym meaning. For multivariate “multivariate metal‐organic framework” OR “multivariate MOF” OR “multivariate reticular” and for flexibility, “metal‐organic framework” AND “flexibility” OR “flexible” was searched.

## Conflict of Interest

The authors declare no conflict of interest.
